# Differentiation of recurrent glioblastoma from radiation necrosis using diffusion radiomics with machine learning model development and external validation

**DOI:** 10.1038/s41598-021-82467-y

**Published:** 2021-02-03

**Authors:** Yae Won Park, Dongmin Choi, Ji Eun Park, Sung Soo Ahn, Hwiyoung Kim, Jong Hee Chang, Se Hoon Kim, Ho Sung Kim, Seung-Koo Lee

**Affiliations:** 1 Department of Radiology and Research Institute of Radiological Science and Center for Clinical Image Data Science, Yonsei University College of Medicine, 50-1 Yonsei-ro, Seodaemun-gu, Seoul, 120-752 South Korea; 2 Department of Computer Science, Yonsei University, Seoul, South Korea; 3 Department of Radiology and Research Institute of Radiology, University of Ulsan College of Medicine, Seoul, South Korea; 4 Department of Neurosurgery, Yonsei University College of Medicine, Seoul, South Korea; 5 Department of Pathology, Yonsei University College of Medicine, Seoul, South Korea

**Keywords:** Cancer, Machine learning, Diagnostic markers

## Abstract

The purpose of this study was to establish a high-performing radiomics strategy with machine learning from conventional and diffusion MRI to differentiate recurrent glioblastoma (GBM) from radiation necrosis (RN) after concurrent chemoradiotherapy (CCRT) or radiotherapy. Eighty-six patients with GBM were enrolled in the training set after they underwent CCRT or radiotherapy and presented with new or enlarging contrast enhancement within the radiation field on follow-up MRI. A diagnosis was established either pathologically or clinicoradiologically (63 recurrent GBM and 23 RN). Another 41 patients (23 recurrent GBM and 18 RN) from a different institution were enrolled in the test set. Conventional MRI sequences (T2-weighted and postcontrast T1-weighted images) and ADC were analyzed to extract 263 radiomic features. After feature selection, various machine learning models with oversampling methods were trained with combinations of MRI sequences and subsequently validated in the test set. In the independent test set, the model using ADC sequence showed the best diagnostic performance, with an AUC, accuracy, sensitivity, specificity of 0.80, 78%, 66.7%, and 87%, respectively. In conclusion, the radiomics models models using other MRI sequences showed AUCs ranging from 0.65 to 0.66 in the test set. The diffusion radiomics may be helpful in differentiating recurrent GBM from RN.

.

## Introduction

The current gold standard treatment for glioblastoma (GBM, World Health Organization [WHO] grade IV) is maximum safe tumor resection, followed by concurrent chemoradiotherapy (CCRT) with temozolomide^1,2^. In cases of elderly patients with unmethylated 6-methylguanine-DNA methyltransferase (MGMT) promoter status or patients with Karnofsky performance status (KPS) index lower than 70, radiotherapy (RT) alone is the standard treatment^2,3^. Radiation necrosis (RN) usually occurs within 3 years after radiation therapy and is often indistinguishable from recurrent tumor because it manifests as an enhancing mass lesion with varying degrees of surrounding edema and progressive enhancement on serial magnetic resonance imaging (MRI)^4,5^. Thus, distinguishing between recurrent GBM and RN has clinical importance in deciding the subsequent management; recurrence indicates treatment failure and requires the use of additional anticancer therapies, whereas RN is treated conservatively.

Multiple studies have made efforts to distinguish GBM recurrence from RN using various imaging methods, including conventional imaging, diffusion-weighted imaging (DWI), diffusion tensor imaging, dynamic susceptibility contrast (DSC) imaging, MR spectroscopy, amide proton transfer imaging, and positron emission tomography^4–13^. However, there is no gold standard imaging method for the differentiation between recurrence and RN, due to high degree of overlapping findings. Currently, the definitive diagnosis is based on histopathology which is both invasive and difficult. In addition, the pathology results may be variable depending on the surgical sampling sites due to the coexistence and admixture of recurrence and RN^14^.

Radiomics involves the identification of ample quantitative features within images and the subsequent data mining for information extraction and application^15^. Recent studies have shown promising results in predicting the molecular status, grade, and prognosis of gliomas^16–20^. Because radiomics models use high-throughput features, there are prone to discover invisible information which are inaccessible with single-parameter analysis.

The aim of this study was to develop and validate a high-performing radiomic strategy using machine learning classifiers from conventional imaging and apparent diffusion coefficient (ADC) to differentiate recurrent GBM from RN after concurrent CCRT or radiotherapy.

## Results

### Baseline characteristics of the patients

The baseline demographic and clinical characteristics are summarized in Table 1. Of the 86 patients in the training set, 63 (73.3%) were classified as recurrent GBM and 23 (26.7%) as RN cases. The 41 patients in the test set consisted of 23 (56.1%) recurrent GBM and 18 (43.9%) RN cases. There were no significant differences in age, sex, extent of resection, first line treatment (either CCRT or RT alone/RT plus temozolomide), total radiation dose, isocitrate dehydrogenase 1 (IDH1) mutation status, and MGMT methylation status between patients with recurrent GBM and those with RN within both training and test sets.

**Table 1:** Baseline demographic data and clinical characteristics of patients.

Variables	Training set (n = 86)			Test set (n = 41)			P-value^b^
	Recurrent GBM	RN	P-value^a^	Recurrent GBM	RN	P-value^a^	
Patient no	63	23	–	23	18	–	
Age (years)	54.4 ± 13.0	57.9 ± 10.6	0.255	60.7 ± 11.8	57.0 ± 13.7	0.358	0.571
Female sex	20 (31.7)	7 (30.4)	0.908	10 (43.5)	8 (44.4)	0.951	0.851
KPS	73.8 ± 17.0	73.9 ± 19.2	0.974	60.7 ± 11.8	57.0 ± 13.7	0.394	0.961
Extent of resection			0.644			0.556	0.757
Biopsy	7 (11.1)	1 (4.3)		1 (4.3)	1 (5.6)		
Partial	11 (17.5)	5 (21.7)		7 (30.4)	3 (16.7)		
Subtotal	24 (38.1)	11 (47.8)		13 (56.5)	10 (55.6)		
Total	21 (33.3)	6 (26.1)		2 (8.7)	4 (22.2)		
First-line treatment			0.3115			0.370	0.418
CCRT	60 (93.7)	20 (87.0)		22 (95.7)	17 (94.4)		
RT alone or RT plus temozolomide	4 (6.3)	3 (13.0)		1 (4.3)	1 (5.6)	0.859	
Total radiation dose (Gy)	60.2 ± 11.6	61.9 ± 16.1	0.591	56.3 ± 11.4	60.7 ± 8.1	0.251	0.476
IDH1 mutant	2 (3.2)	2 (8.7)	0.282	1 (4.3)	1 (5.6)	0.859	0.342
MGMT promoter methylation	13 (20.6)	9 (39.1)	0.082	8 (34.8)	7 (38.9)	0.786	0.090

*GBM *glioblastoma, *RN *radiation necrosis, *KPS *Karnosfky performance status, *MGMT *oxygen 6-methylguanine DNA methyltransferase.

Data are presented as either mean ± standard deviation or numbers of patients (%).

^a^Calculated from Student t test for continuous variables and Chi-square test for categorical variables for comparison of recurrent GBM and RN in training and test sets.

^b^Calculated from Student t test for continuous variables and Chi-square test for categorical variables for comparison of training and test sets.

### Qualitative imaging analysis

The radiologists’ assessment of conventional imaging features showed no significant difference between recurrent GBM and RN in maximum lesion diameter, involvement of corpus callosum, and “Swiss cheese” or “spreading wavefront” enhancement pattern in both the training set and test sets (all p-values > 0.05), respectively.

### Best performing machine learning models from radiomics features for differentiating recurrent GBM from RN in the training set

Using radiomic features, in each combination of the selected MRI sequence, the 3 feature selection, 3 classification methods, and 2 oversampling methods were trained.

**Figure 1:**
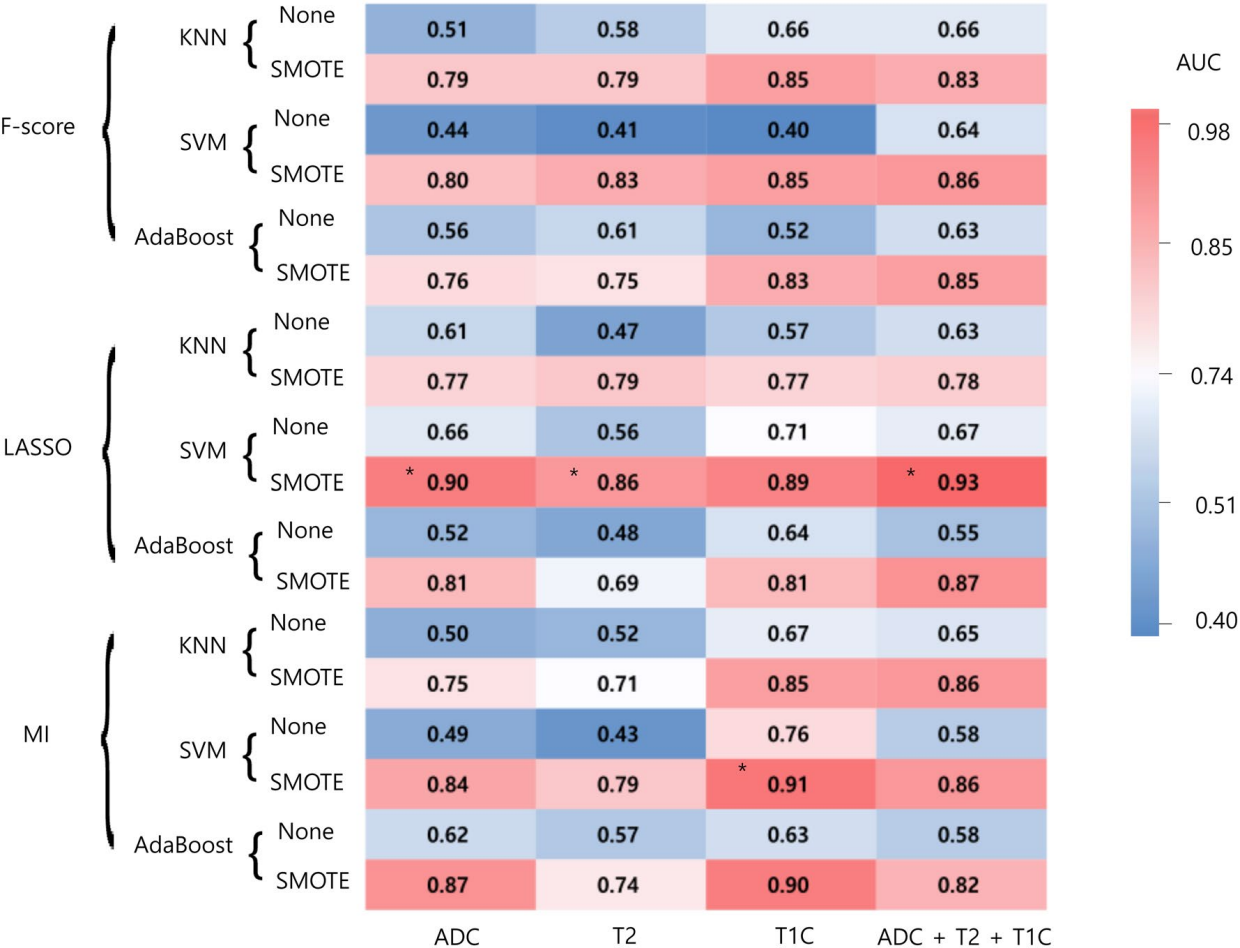
Heatmap depicting the diagnostic performance (AUCs) of combinations of feature selection methods, classifiers, and combination of sequences in the training set. *AUC *area under the curve, *KNN *k-nearest neighbors, *MI *mutual information, *LASSO *least absolute shrinkage and selection operator, *SMOTE *synthetic minority over-sampling technique, *SVM *support vector machine, *T1C *postcontrast T1WI, *T2 *T2WI. The best performing model in each combination of MRI sequence and mask are marked in asterisks (*).

The performance of each combination of the models is shown in Fig. 1. In the training set, the area under the curve (AUCs) of the models showing the best diagnostic performance ranged from 0.86 to 0.93 in each combination. AUCs with oversampling were higher than those without oversampling in all combinations. In the ADC sequence, the combination of least absolute shrinkage and selection operator (LASSO) feature selection, and support vector machine (SVM) showed the best diagnostic performance in the training set. The selected 18 features consisted of 3 first-order features, 10 s-order features, and 5 shape features (Detailed information at Supplementary Table 3). This model demonstrated an area under the curve (AUC), accuracy, sensitivity, specificity of 0.90 (95% confidence interval [CI] 0.84–0.95), 80.5%, 78.3%, and 82.9%, respectively. In the T2WI (T2) sequence, the combination of LASSO feature selection and SVM showed the best diagnostic performance in the training set with an AUC of 0.86 (95% CI 0.80–0.91). In the postcontrast T1WI (T1C) sequence, the combination of mutual information (MI) feature selection and SVM showed the best diagnostic performance in the training set with an AUC of 0.91 (95% CI 0.86–0.95). In the combined sequence (ADC + T2 + T1C), the combination of LASSO feature selection, and SVM showed the best diagnostic performance in the training set with an AUC of 0.93 (95% CI 0.89–0.97). (Hyperparameters for each model are summarized at Supplementary Table 4).

### Robustness of radiomics models in the test set

In the independent test set, the model using ADC sequence with the combination of LASSO feature selection and SVM showed the best diagnostic performance. This model demonstrated an AUC, accuracy, sensitivity, specificity of 0.80 (95% CI 0.65–0.95), 78%, 66.7%, and 87%, respectively.

The radiomics models using other combination of MRI sequence showed poor performance (AUCs ranging from 0.65 to 0.66) in the test set, although it did not reach significant difference from the ADC radiomics model (p-values of > 0.05). Table 2 summarizes the results of best performing models in training and test sets.

**Table 2:** Diagnostic performance of the best performing machine learning model in the training set and the test set.

Sequence	Feature selection	No. of selected features	Classification	Training set					Test set				
				AUC (95% CI)	Accuracy (95% CI)	Sensitivity (95% CI)	Specificity (95% CI)	P-value*	AUC (95% CI)	Accuracy (95% CI)	Sensitivity (95% CI)	Specificity (95% CI)	P-value*
ADC	LASSO	18	SVM	0.90 (0.84–0.95)	80.5 (77.4–83.6)	78.3 (64.2–92.4)	82.9 (74.7–91.1)	Reference	0.80 (0.65–0.95)	78.0 (62.4–89.4)	66.7 (41.0–86.7)	87.0 (66.5–97.2)	Reference
T2	LASSO	21	SVM	0.86 (0.80- 0.91)	77.1 (74.1–80.1)	80.7 (70.8–90.6)	73.1 (66.0–80.2)	0.346	0.65 (0.48–0.82)	61.0 (44.5–75.8)	44.4 (21.5–69.2)	73.9 (51.6–89.9)	0.186
T1C	MI	30	SVM	0.91 (0.86–0.95)	87.4 (84.5–90.3)	90.7 (83.0–98.4)	84.3 (78.2–90.4)	0.798	0.66 (0.49–0.83)	53.7 (37.4–69.3)	11.1 (1.4–34.7)	87.0 (66.4–97.2)	0.217
ADC + T2 + T1C	LASSO	35	SVM	0.93 (0.89–0.97)	85.2 (82.0–88.4)	79.8 (71.2–88.4)	90.5 (83.0–98.0)	0.405	0.66 (0.49–0.84)	63.4 (46.9–77.9)	38.9 (17.3–64.3)	82.6 (61.2–95.0)	0.217

All training set performance was calculated on SMOTE generated datasets.

*CI *confidence interval, *LASSO *least absolute shrinkage and selection operator, *MI *mutual information, *SMOTE *synthetic minority over-sampling technique, *SVM *support vector machine, *T1C *postcontrast T1WI, *T2 *T2WI.

*P-value refers to the significance among the differences of the AUCs between the ADC radiomics model and the other models.

## Discussion

In this study, we evaluated the ability of conventional and diffusion radiomics to differentiate recurrent GBM from RN. Several MR sequences and their combination were investigated and validated externally, and among these models the diffusion radiomics model showed robustness with AUC of 0.80. RN has been reported to occur in approximately 9.8–44.4% of treated gliomas, which shows low incidence than recurrent GBM^6,9,21^. In our study, the data imbalance was mitigated by using a systematic algorithm, which generates synthetic samples in the minority class^22^. The performance was increased when synthetic minority over-sampling technique (SMOTE) was applied in our dataset (Fig. 1), showing its efficacy. Although recurrent GBM and RN have similar radiologic appearances, they harbor distinct radiomic information that can be extracted and used to build a clinically relevant predictive model that discriminates recurrent GBM from RN. Our model may aid in deciding the subsequent management of these patients.

Although conventional findings such as “Swiss cheese” or “spreading wavefront” enhancement pattern have been reported to show differences between recurrent high-grade glioma and RN in earlier studies^5,6^, these findings have subsequently been reported that they cannot be reliably used alone in differentiating between the two conditions^4,23^. Moreover, these conventional imaging patterns are highly subjective. Various studies implementing advanced imaging parameters such as diffusion MRI, DSC MRI, proton MR spectroscopy (MRS), amide proton transfer (APT) imaging, and positron emission tomography (PET) have shown promising results in differentiating recurrent GBM from RN^9,11,12,24–26^. Although APT imaging has shown higher diagnostic performance than MRS^27^ or ^11^C-MET PET^28^ in differentiating recurrent GBM from RN, APT imaging is challenging due to long scan times and limited coverage with high radiofrequency power. On the other hand, the accuracy of MRS and PET in differentiating recurrent GBM from RN has been questioned; a meta-analysis has shown moderate sensitivity and specificity for MRS, ^18^F-FDG, and ^11^C-MET PET in distinguishing between recurrent GBM from RN^29^, whereas another study found no difference between recurrence and necrosis groups using ^18^F-FDG and ^11^C-MET PET^12^. MRS and PET also have limited value in practical clinical settings due to their limited availability and low cost-effectiveness. DSC MRI can readily distinguish between recurrent GBM and RN, as a biomarker of angiogenesis, with higher availability^9,30^. However, the relative cerebral blood volume from DSC MRI can produce false positive or false negative results due to volume averaging, susceptibility artifacts, and overlapping portions in RN and recurrent GBM^4,31^. Also, the optimal thresholds are different depending on the specific protocol^9,32^, and values derived from DSC imaging are relative values compared to absolute values from ADC maps. Moreover, the previous studies using advanced imaging focused on single parameters such as mean values.

In contrast to extraction of single parameters, radiomics extracts high-throughput quantitative features within the regions of interest and has been reported to be a potentially useful approach for estimating the molecular status, grade, and prognosis of brain tumors^16,17,19,20,33,34^. Previous studies have showed promising results in identifying recurrent brain tumor from RN using radiomics^35–37^. However, these studies were focused on recurrent brain metastases rather than recurrent GBM, analyzing only conventional MRI sequences, and most datasets were small without external validation. Recent studies implemented radiomics model in differentiating recurrent glioma from RN^38,39^; however the studies was either performed in a smaller dataset without external validation using only conventional MRI^38^, or performed radiomics analysis using ^18^F-FDG and ^11^C-MET PET^39^, which are not routinely acquired imaging modalities. Our radiomics model implemented not only conventional MRI but also ADC map, which are recommended sequences in the glioma protocol^40,41^, and showed that diffusion radiomics model could robustly differentiate recurrent GBM from RN better than any other radiomics model. However, models using conventional MRI sequences (such as T2 or T1C) showed AUCs ranging from 0.650 to 0.662 in the test set. Moreover, multiparametric radiomics model did not show increased performance than the diffusion radiomics model in the external validation. The signal intensities in conventional images may differ in different MRI protocol settings, leading to poor performance in an external validation even after signal intensity normalization. On the other hand, ADC maps extract absolute values creating reliable feature extraction, which may be less affected by heterogeneous protocol settings and consequently demonstrated high diagnostic performance in the external validation. In addition, our results may emphasize the importance of domain-specific knowledge in the relatively small data settings of radiomics study^42^. Previous studies have shown that the ADC characteristics are more important than conventional characteristics in differentiating RN from GBM^4,7^. The diffusion radiomics model is promising for reflecting the tumor microenvironment, since these values can contain biological information^43,44^. Although ADC value can be affected by various factors, ADC in tumor is generally considered to be an index of tumor cellularity that reflects tumor burden^45,46^. On histopathological examination, recurrent GBM is characterized by dense glioma cells, which limit water diffusion^7^. In contrast, RN is characterized by extensive fibrinoid necrosis, vascular dilatation, and gliosis^47^. The different histopathology and spatial complexity may be reflected in diffusion radiomics, allowing the differentiation of the two entities^31^.

In our study, the majority of significant radiomics features from the diffusion radiomics model were various second-order features, suggesting that high‐throughput characteristics can provide more accurate assessment. The hypothesis for this observation is that second-order features capture the spatial variation in signal intensity, which tend to extract information that may be incomprehensible and invisible to the naked eye. Recent studies have demonstrated that second-order features also reflect the underlying histology^48,49^. However, a future study with histopathologic correlation is mandatory to prove our hypothesis of the direct relationship between radiomic features in recurrent GBM and RN. Various features such as flatness, sphericity, mesh volume, and major axis length were included, suggesting that the quantitative shape features may aid in differentiating in recurrent GBM from RN. Because there was no previous study that has quantified various shape features from the whole 3D lesion, further studies are indicated to validate our results.

Our study has several limitations. First, our study was retrospective with a small data size. Due to the relatively small size of the test set, the 95% CIs of the AUCs in the test set tended to have a large range and some 95% CIs of the radiomics models cross 0.5. Future studies should be performed with a larger dataset. Second, DSC imaging was not included due to lack of data in a portion of patients. Because DSC data is important in distinguishing recurrent GBM from RN^50^, further radiomics studies implementing DSC data are warranted to evaluate the efficacy. Third, fluid-attenuation inversion recovery (FLAIR) sequence was not utilized in this study due to mixture of both precontrast and postcontrast FLAIR sequences in the training set. Further studies are warranted to include the FLAIR sequence in radiomics analysis. Fourth, clinical factors were not integrated into the radiomics model due to statistical insignificance in our dataset. However, as previous studies have stated the relationship between radiation doses or fractionation schemes with RN^51,52^, future radiomics studies with larger datasets should perform multivariable analysis with clinically relevant features to differentiate recurrent GBM from RN. Fifth, cross-validation was performed separately in the feature selection stage and the machine learning classification stage, which may have led to overfitted results.

In conclusion, the diffusion radiomics model may be helpful in differentiating recurrent GBM from RN.

## Methods

### Patient population

The Yonsei University Institutional Review Board waived the need for obtaining informed patient consent for this retrospective study. All methods were carried out in accordance with relevant guidelines and regulation. For research limited to patients' medical records, access was cleared by the Yonsei University Institutional Review Board and was supervised by a person (S-K.L.) who was fully aware of the confidentiality requirements. All of the study protocols were approved by the Institutional Review Board (Severance Hospital, Yonsei University Health System Institutional Review Board, 2018-1472-002). Between February 2016 and February 2019, 90 patients with pathologically diagnosed GBM (WHO grade IV) from our institution were reviewed in this study. The inclusion criteria were as follows: (1) GBM confirmed by histopathology; (2) postoperative CCRT or RT, with a radiation dose ranging from 45 to 70 Gy; (3) subsequent development of a new or enlarging region of contrast enhancement within the radiation field 12 weeks after CCRT or RT; and (4) surgical resection of the enhancing lesion or adequate clinicoradiological follow-up, which enabled us to diagnose recurrent GBM or RN. For clinicoradiological diagnosis, a final diagnosis of recurrent GBM was made if the contrast-enhancing lesions gradually enlarged on more than two subsequent follow-up MRI studies performed at 2–3 month intervals (with a size criterion of an increase of > 25% of the size of a measurable [> 1 cm] enhancing lesion according to the sum of the products of perpendicular dimensions) and the clinical symptoms of patients showed gradual deterioration during follow-up^28^. Alternatively, a final diagnosis of RN was made if enhancing lesions gradually decreased on more than two subsequent follow-up MRI studies performed at 2–3 month intervals and clinical symptoms improved during the follow-up period. Exclusion criteria were as follows: (1) processing error (n = 3), (2) absence of MRI sequences (n = 1). Thus, a total of 86 patients were enrolled.

Identical inclusion and exclusion criteria were applied and 41 patients from another institutional hospital (Asan Medical Center, Seoul, Korea) were enrolled in the test set. The clinical characteristics of the patients included age, sex, KPS, IDH mutational status, MGMT promoter methylation status, and the extent of resection of the tumor (gross total resection, subtotal resection, partial resection, or biopsy).

### Pathological diagnosis

All patients underwent initial surgery, and histologic confirmation was obtained according to the 2016 WHO classification^46^. Peptide nucleic acid-mediated clamping polymerase chain reaction and immunohistochemical analysis were performed to detect the R132H mutation status in IDH1^53^. MGMT promoter methylation status was diagnosed on the basis of methylation-specific polymerase chain reaction^54^.

Twenty-two and 14 patients underwent second-look operations in the training set and test set, respectively. In second-look operations, the pathological diagnoses included 17 recurrent GBM and 5 RN cases in the training set, and 8 recurrent GBM and 6 RN cases in the test set, respectively. The diagnosis was made on the basis of histological findings in contrast-enhancing tissue obtained with surgical tumor resection or image-guided. More than 5% viable tumor diagnosed during the histological examination by neuropathologists, were classified as a recurrent GBM^9^.

### MRI protocol

In the training set, all patients underwent MRI on a 3.0-T MRI scanner (Achieva or Ingenia, Philips Medical Systems) with an 8-channel head coil. The preoperative MRI sequences included T1WI, T2, T1C, as well as ADC scans. After 5–6 min of administration of 0.1 mL/kg of gadolinium-based contrast material (Gadovist; Bayer), T1C were acquired.

In the external validation set, MRI exams were performed using a 3.0-T MRI scanner (Achieva, Philips Medical Systems) with an 8-channel head coil. Scaling and un-normalization of ADC pixel values generated at the scanner was performed as previously described^55^. Constant level appearance (CLEAR) processing, a technique to achieve homogeneity correction by using coil sensitivity maps acquired in the reference scan, was performed^55^. The acquisition protocols are described in further details in the Supplementary Table 1.

### Qualitative image analysis

Conventional images were analyzed by two neuroradiologists (with 14 years and 7 years of experience) for maximum lesion diameter, involvement of corpus callosum, and “Swiss cheese” or “spreading wavefront” (ill-defined margins of the enhancement) enhancement pattern, according to previous literature^5,6^. Discrepancies were settled by consensus.

### Image preprocessing and radiomics feature extraction

Preprocessing of T2, T1C images, and ADC map was performed to standardize the data analysis among patients. Low-frequency intensity nonuniformity was corrected by applying the N4 bias correction algorithm as implemented in the Advanced Normalization Tools (ANTs)^56^. Signal intensity normalization was used to reduce variance in the T2 and T1C images, by applying the WhiteStripe method from R package^57^. T2, T1C, and ADC images were resampled to a uniform voxel size of 1 × 1 × 1 mm. T2 and ADC images were registered to the T1C image using affine transformation with normalized mutual information as a cost function. Tumor segmentation was performed through a consensus discussion of two neuroradiologists (with 14 years and 7 years of experience), in order to select the contrast-enhancing solid portion of the tumor on T1C images. Segmentation was performed semiautomatically with an interactive level-set region of interest, using edge-based and threshold-based algorithms using 3D Slicer (version 4.11.0). There was no distortion in the ADC images that affected the segmented masks. Radiomic features were extracted from the segmented mask, with a bin size of 32, with an open-source python-based module (PyRadiomics, version 2.0)^58^, which was adherent to the Image Biomarker Standardization Initiative (IBSI) guideline^59^. A total of 93 radiomic features, including shape, first order features, and second-order features (Supplementary Table 2), were extracted from the mask. In addition, edge contrast calculation was performed, that characterizes the tumor border, as previously described (Supplementary Information S1)^60^. The final set consisted of 263 radiomic features (14 shape features + 83 first-order and second-order 14 features × 3 sequences) for each patient. The data were processed using a multi-platform, open-source software package (3D slicer, version 4.6.2-1; http://slicer.org).

### Statistical analysis

Baseline characteristics were compared between recurrent GBM and RN patients using chi-squared or Fisher’s exact test for categorical variables, independent *t*-tests for normally distributed continuous variables, and Mann–Whitney *U*-tests for continuous variables without normal distribution. DeLong’s method was used to compare the AUCs among the ADC radiomics model and other radiomics models in the training and test sets^61^. Statistical significance was set at P < 0.05.

### Radiomic feature selection and machine learning

**Figure 2:**
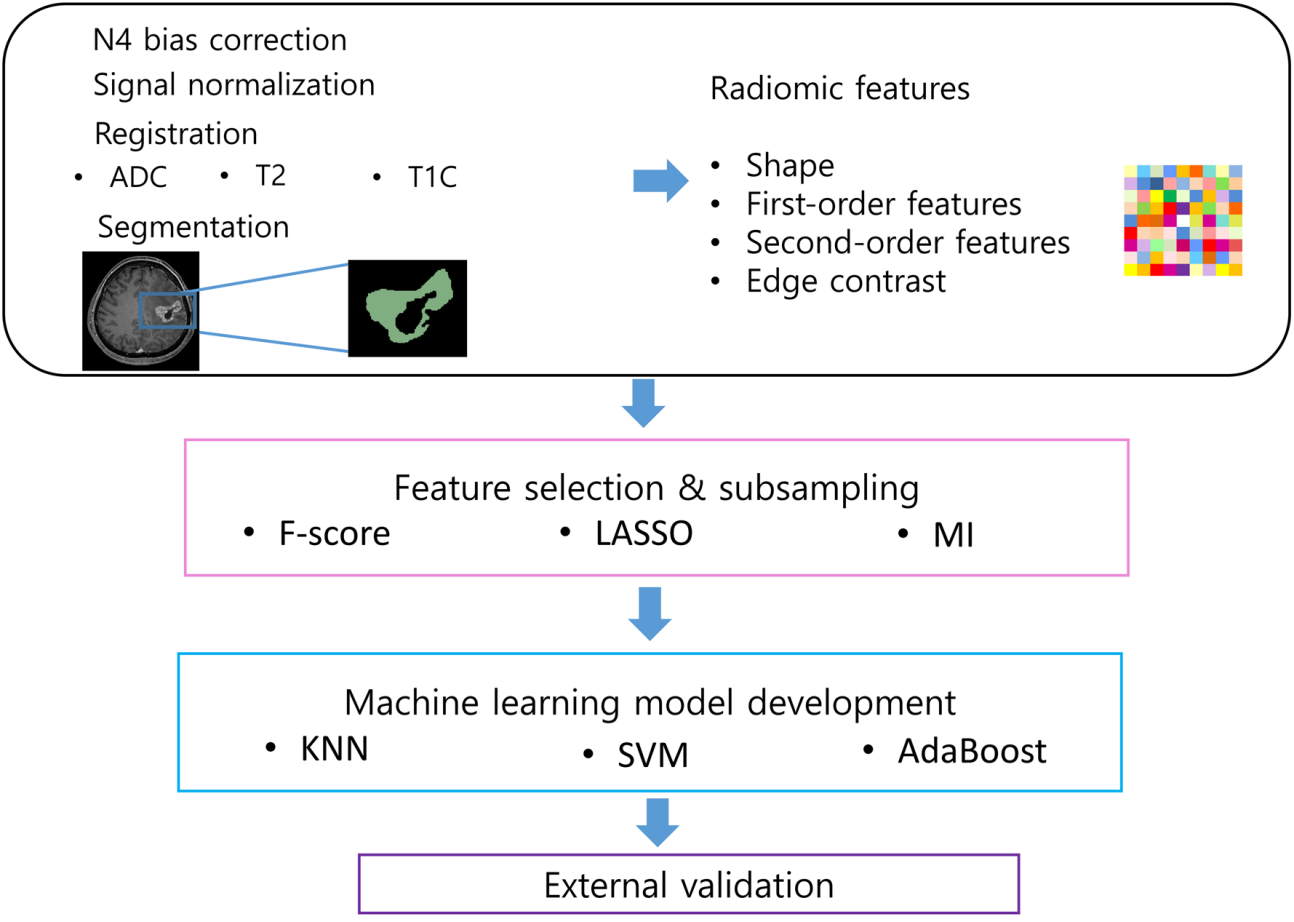
The radiomics pipeline of our study. *KNN *k-nearest neighbors, *MI *mutual information, *LASSO *least absolute shrinkage and selection operator, *SVM *support vector machine, *T1C *postcontrast T1WI, *T2 *T2WI.

The schematic of the radiomics pipeline is shown in Fig. 2. All radiomic features were normalized using z-score normalization. For feature selection, the F-score, LASSO, or MI with stratified ten-fold cross-validation were applied^62^. After feature selection, the machine learning classifiers were constructed separately using k-nearest neighbors (KNN), SVM, or AdaBoost, with stratified ten-fold cross-validation. The optimal hyperparameters producing the highest AUC were selected by random search during cross-validation and subsequently used to get the final model. In addition, to overcome data imbalance, each machine learning model was trained either without oversampling or with SMOTE (with a 1:1 ratio)^22^. Because we wanted to determine which combination of MRI sequence shows the highest performance, the identical process was performed in each sequence (ADC, T2, T1C, and combined ADC, T2, and T1C model). Thus, various combinations of classification models were trained to differentiate recurrent GBM from RN in the training set. AUC, accuracy, sensitivity, and specificity were obtained in the SMOTE generated dataset in the training set, with a cutoff value according to Youden’s index. The different feature selection, classification methods, and oversampling were computed using MatlabR2014b (Mathworks). Statistical significance was set at P < 0.05.

### Diagnostic performance in the test set

Based on the radiomics classification model in the training set, the best combination of feature selection, classification methods, and oversampling in each sequence was used in the test set. The AUC, accuracy, sensitivity, and specificity were obtained with the same cutoff from the training set.

## Supplementary Information

Supplementary Information.

## Abbreviations

ADC: Apparent diffusion coefficient
APT: Amide proton transfer
CI: Confidence interval
DWI: Diffusion-weighted imaging
DSC: Dynamic susceptibility contrast
IDH1: Isocitrate dehydrogenase1
KNN: K-nearest neighbors
KPS: Karnofsky performance status
LASSO: Least absolute shrinkage and selection operator
MGMT: Oxygen 6-methylguanine-DNA methyltransferase
MI: Mutual information
MRI: Magnetic resonance imaging
MRS: Magnetic resonance spectroscopy
PET: Positron emission tomography
SMOTE: Synthetic minority over-sampling technique
SVM: Support vector machine
T1C: Postcontrast T1WI
T2: T2WI
GBM: Glioblastoma
RN: Radiation necrosis
RT: Radiation therapy
