# Supplementary material for: Differentiation of recurrent glioblastoma from radiation necrosis using diffusion radiomics with machine learning model development and external validation
Source: Sci Rep. 2021 Feb 3;11:2913. doi: 10.1038/s41598-021-82467-y (PMC7858615; doi:10.1038/s41598-021-82467-y)
Supplement: Supplementary file 1 — Supplementary Information. [file 41598_2021_82467_MOESM1_ESM.docx]

**Supplementary Material**

**S1. Edge contrast (EG)**

EC is defined as the gradient magnitude of the lesion edges, where higher EC indicates a sharper and more distinct border compared to lower EC. The following process was applied to the T2, T1C, and ADC images to extract the lesion surfaces and calculate the EC, as previously described. [1] 3D analyses were performed to decrease the partial volume effect and increase the local precision.

First, morphologic operations of erosion and dilation were applied to the mask using the spherical 3D mask to remove the holes and small islands. Then, the contour of the mask was extracted in 3D, indexing the surface of the regions of interest. The gradients of the images were calculated and were overlaid on the surface of the 3D binary mask to create the hyperintense surface with the initial EC. The initial EC was modified by removing the pixels with the highest 10% intensity to minimize the effect of edge magnitude arising from the CSF and skull.

A total of four EC parameters were calculated for each edge magnitude layer in each mask per sequence; EC100% = average magnitude of all points on the edge, EC75% = average magnitude of the lowest 75% of points, EC50% = average magnitude of the lowest half of points, and EC25% = average magnitude of the lowest 25% of points.

Supplementary Table 1. Comparison of MR imaging acquisition protocol of the training and test sets at the institution.

| Pulse Sequence | Training set | Test set |
| --- | --- | --- |
| T1WI |  |  |
| Repetition Time (ms) | 2000 | 2000 |
| Echo Time (ms) | 10 | 20 |
| Matrix | 256 × 256 | 256 × 256 |
| Section thickness (mm) | 5 | 5 |
| Field of view (mm) | 220-240 | 230-240 |
| T2WI |  |  |
| Repetition Time (ms) | 3000 | 3000 |
| Echo Time (ms) | 80 | 80 |
| Matrix | 256 × 256 | 256 × 256 |
| Section thickness (mm) | 1-2 | 5 |
| Field of view (mm) | 220-240 | 230-240 |
| T1C |  |  |
| Repetition Time (ms) | 6.3 | 9.8 |
| Echo Time (ms) | 3.60 | 4.6 |
| Matrix | 256 × 256 or 192 × 192 | 512 × 512 |
| Section thickness (mm) | 1-2 | 1 |
| Field of view (mm) | 220-240 | 256 |
| DWI |  |  |
| b-values (s/mm^2^) | 0,1000 | 0,1000 |
| Repetition Time (ms) | 8400-8500 | 3000–4000 |
| Echo Time (ms) | 70-80 | 56–61.7 |
| Matrix | 256 × 256 | 256 × 256 |
| Section thickness (mm) | 2 | 4–5 |
| Field of view (mm) | 240 | 250 |
| Acceleration factor | 2.5 | N/A |
| Acquisition time | 3 min 14 s | 39 s |

Note: T1C = postcontrast T1WI, DWI = diffusion weighted imaging, N/A = not applied

Supplementary Table 2. List of radiomics features.

| Feature category | Feature list |
| --- | --- |
| Shape (n = 14) | Volume, surface area, surface area to volume ratio, sphericity, maximum 3D diameter, maximum 2D diameter (column), maximum 2D diameter (row), maximum 2D diameter (slice), major axis, minor axis, least axis, elongation, flatness, mesh volume |
| First-order statistics (n = 18) | Energy, total energy, entropy, minimum, 10^th^ percentile, 90^th^ percentile, maximum, mean, median, interquartile range, range, mean absolute deviation, robust mean absolute deviation, root mean squared, skewness, kurtosis, variance, uniformity |
| GLCM (n = 24) | Autocorrelation, cluster prominence, cluster shade, cluster tendency, contrast, correlation, difference average, difference entropy, difference variance, inverse difference, inverse difference moment, inverse difference moment normalized, inverse difference normalized, informal measure of correlation 1, informal measure of correlation 2, inverse variance, joint average, joint energy, joint entropy, maximal correlation coefficient, maximum probability, sum average, sum entropy, sum of squares |
| GLRLM (n = 16) | Short-run emphasis, long-run emphasis, gray level nonuniformity, gray level nonuniformity normalized, run-length nonuniformity, run-length nonuniformity normalized, run percentage, gray level variance, run variance, run entropy, low gray level run emphasis, high gray level run emphasis, short-run low gray level emphasis, short-run high gray level emphasis, long-run low gray level emphasis, long-run high-gray level emphasis |
| GLSZM (n = 16) | Small area emphasis, large area emphasis, gray level non-uniformity, gray level non-uniformity normalized, size-zone non-uniformity, size-zone non-uniformity normalized, zone percentage, gray level variance, zone variance, zone entropy, low gray level zone emphasis, high gray level zone emphasis, small area low gray level emphasis, small area high gray level emphasis, large area low gray level emphasis, large area high gray level emphasis |
| NGTDM (n = 5) | Coarseness, complexity, strength, contrast, busyness |
| Edge contrast (n = 4) | EC100%, EC75%, EC50%, EC25% |

GLCM = gray level co-occurrence matrix, GLRLM = gray level run-length matrix, GLSZM = gray level size zone matrix, NGTDM = neighboring gray tone difference matrix, EC = edge contrast

The details of feature calculation are described at the Pyradiomics Site (https://pyradiomics.readthedocs.io/en/latest/features.html).

Supplementary Table 3. Selected features from the LASSO procedure for the best performing model using ADC sequence and CE mask.

| Feature category | Feature name |
| --- | --- |
| first-order statistics | kurtosis |
| First-order statistics | mean absolute deviation |
| First-order statistics | range |
| GLCM | inverse difference normalized |
| GLCM | informal measure of correlation 1 |
| GLCM | informal measure of correlation 2 |
| GLRLM | run variance |
| GLSZM | gray level non-uniformity |
| GLSZM | low gray level zone emphasis |
| GLSZM | size zone non-uniformity |
| GLSZM | small area high gray level emphasis |
| NTGDM | complexity |
| NTGDM | strength |
| Shape | flatness |
| Shape | major axis length |
| Shape | maximum 2D diameter (slice) |
| Shape | mesh volume |
| Shape | sphericity |

ADC = apparent diffusion coefficient, CE = contrast enhancing, GLCM = gray level co-occurrence matrix, GLRLM = gray level run-length matrix, GLSZM = gray level size zone matrix, NGTDM = neighboring gray tone difference matrix

Supplementary Table 4. Hyperparameters according to the different combinations of feature selection methods, classifiers, and combinations of sequences in the training set.

| Feature selection method | Classifier | Subsampling | Optimal feature number | Optimal hyperparameters |
| --- | --- | --- | --- | --- |
| ADC sequence | | | | |
| F-score | KNN | None | 29 | K = 2 |
|  |  | SMOTE | 29 | K = 12 |
|  | SVM | None | 29 | C = 9 |
|  |  | SMOTE | 29 | C = 7, gamma = 10 |
|  | AdaBoost | None | 29 | N_estimators = 200, Learning_rate = 1 |
|  |  | SMOTE | 29 | N_estimators = 110, Learning_rate = 0.5 |
| LASSO | KNN | None | 18 | K = 2 |
|  |  | SMOTE | 18 | K = 2 |
|  | SVM | None | 18 | C = 9, gamma = 1 |
|  |  | SMOTE | 18 | C = 3, gamma = 10 |
|  | AdaBoost | None | 18 | N_estimators = 220, Learning_rate = 0.1 |
|  |  | SMOTE | 18 | N_estimators = 110, Learning_rate = 0.01 |
| MI | KNN | None | 29 | K = 2 |
|  |  | SMOTE | 29 | K = 8 |
|  | SVM | None | 29 | C = 5 |
|  |  | SMOTE | 29 | C = 1, gamma = 0.1 |
|  | AdaBoost | None | 29 | N_estimators = 210, Learning_rate = 1 |
|  |  | SMOTE | 29 | N_estimators = 160, Learning_rate = 0.1 |
| T2 sequence | | | | |
| F-score | KNN | None | 29 | K = 11 |
|  |  | SMOTE | 29 | K = 5 |
|  | SVM | None | 29 | C = 3 |
|  |  | SMOTE | 29 | C = 8, gamma = 0.001 |
|  | AdaBoost | None | 29 | N_estimators = 230, Learning_rate = 0.1 |
|  |  | SMOTE | 29 | N_estimators = 380, Learning_rate = 0.01 |
| LASSO | KNN | None | 21 | K = 2 |
|  |  | SMOTE | 21 | K = 2 |
|  | SVM | None | 21 | C = 1 |
|  |  | SMOTE | 21 | C = 3, gamma = 10 |
|  | AdaBoost | None | 21 | N_estimators = 210, Learning_rate = 1 |
|  |  | SMOTE | 21 | N_estimators = 150, Learning_rate = 0.5 |
| MI | KNN | None | 29 | K = 2 |
|  |  | SMOTE | 29 | K = 2 |
|  | SVM | None | 29 | C = 3 |
|  |  | SMOTE | 29 | C = 3, gamma = 1 |
|  | AdaBoost | None | 29 | N_estimators = 110, Learning_rate = 0.1 |
|  |  | SMOTE | 29 | N_estimators = 290, Learning_rate = 0.5 |
| T1C sequence | | | | |
| F-score | KNN | None | 29 | K = 2 |
|  |  | SMOTE | 29 | K = 2 |
|  | SVM | None | 29 | C = 5 |
|  |  | SMOTE | 29 | C = 5, gamma = 0.1 |
|  | AdaBoost | None | 29 | N_estimators = 200, Learning_rate = 0.1 |
|  |  | SMOTE | 29 | N_estimators = 110, Learning_rate = 0.1 |
| LASSO | KNN | None | 20 | K = 7 |
|  |  | SMOTE | 20 | K = 2 |
|  | SVM | None | 20 | C = 5 , gamma = 10 |
|  |  | SMOTE | 20 | C = 7 |
|  | AdaBoost | None | 20 | N_estimators = 80, Learning_rate = 1 |
|  |  | SMOTE | 20 | N_estimators = 230, Learning_rate = 1 |
| MI | KNN | None | 30 | K = 2 |
|  |  | SMOTE | 30 | K = 2 |
|  | SVM | None | 30 | C = 1, gamma = 0.1 |
|  |  | SMOTE | 30 | C = 3, gamma = 0.1 |
|  | AdaBoost | None | 30 | N_estimators = 170, Learning_rate = 0.1 |
|  |  | SMOTE | 30 | N_estimators = 380, Learning_rate = 0.1 |
| ADC + T2 + T1C sequence | | | | |
| F-score | KNN | None | 29 | K = 2 |
|  |  | SMOTE | 29 | K = 2 |
|  | SVM | None | 29 | C = 1 |
|  |  | SMOTE | 29 | C = 3, gamma = 0.1 |
|  | AdaBoost | None | 29 | N_estimators = 170, Learning_rate = 0.1 |
|  |  | SMOTE | 29 | N_estimators = 140, Learning_rate = 0.1 |
| LASSO | KNN | None | 35 | K = 2 |
|  |  | SMOTE | 35 | K = 2 |
|  | SVM | None | 35 | C = 1, gamma = 0.1 |
|  |  | SMOTE | 35 | C = 3 |
|  | AdaBoost | None | 35 | N_estimators = 170, Learning_rate = 1 |
|  |  | SMOTE | 35 | N_estimators = 320, Learning_rate = 1 |
| MI | KNN | None | 29 | K = 7 |
|  |  | SMOTE | 29 | K = 2 |
|  | SVM | None | 29 | C = 1 |
|  |  | SMOTE | 29 | C = 5 |
|  | AdaBoost | None | 29 | N_estimators = 50, Learning_rate = 1 |
|  |  | SMOTE | 29 | N_estimators = 290, Learning_rate = 0.5 |

AUC = area under the curve, KNN = k-nearest neighbors, MI = mutual information, LASSO = least absolute shrinkage and selection operator, SMOTE = synthetic minority over-sampling technique, SVM = support vector machine, T1C = postcontrast T1WI, T2 = T2WI

**References**

1 Bahrami N, Piccioni D, Karunamuni R et al (2018) Edge contrast of the FLAIR hyperintense region predicts survival in patients with high-grade gliomas following treatment with bevacizumab. American Journal of Neuroradiology 39:1017-1024
